# The combination of serum oligosaccharide chain (G-test), alpha-fetoprotein, and aspartate aminotransferase to alanine aminotransferase ratio provides the optimal diagnostic value for early detection of hepatocellular carcinoma

**DOI:** 10.1186/s12885-022-10139-9

**Published:** 2022-10-14

**Authors:** Wentao Zhu, Pei Shi, An Liang, Ying Zhu, Jiwei Fu, Songsong Yuan, Xiaoping Wu

**Affiliations:** Department of Infectious Diseases, the First Affiliated Hospital of Nanchang University, Nanchang, China

**Keywords:** Hepatocellular carcinoma, Chronic hepatitis, Liver cirrhosis, G-test, Alpha-fetoprotein, Aspartate aminotransferase to alanine aminotransferase ratio, Serological markers, Receiver operating characteristic curve

## Abstract

**Background:**

The purpose of this study was to compare the diagnostic value of serum oligosaccharide chain (G-test), alpha-fetoprotein (AFP) and aspartic aminotransferase to alanine aminotransferase ratios (AAR), both alone and in combination, for predicting hepatocellular carcinoma (HCC) onset.

**Methods:**

Between Januarys 2020–2022, 152 subjects admitted to the First Affiliated Hospital of Nanchang University was enrolled in this study, of which 77 had HCC, 18 chronic hepatitis (CH), 37 liver cirrhosis (LC) and 20 were healthy. Data for patient characteristics were collected, and differences between groups were analyzed by either Mann-Whitney U or χ2 tests. Receiver operating characteristic (ROC) curve analysis was used to determine the diagnostic value of AFP, G-test, and AAR for HCC.

**Results:**

G-test, AFP, and AAR were all found to have close correlations with HCC among the different patient groups, with G-test being the most predictive for HCC among healthy and CL patients, as represented by respective areas under the curve (AUC) of 0.953 and 0.792 (P < 0.001). By contrast, AAR had the greatest diagnostic ability for HCC among CH patients (AUC = 0.850; P < 0.001). However, the combination of all 3 biomarkers obtained the most optimal results for predicting HCC onset, in terms of predictive capability for all 3 non-HCC patient groups, yielding AUCs of 0.958, 0.898, and 0.808 (P < 0.001) for, respectively, healthy, CH, and LC patients. Additionally, AFP had higher specificity, but lower sensitivity, with increased threshold values, as the recommended threshold of AFP ≥ 400 ng/mL yielded a missed diagnosis rate of 72.7%. For AFP-negative HCC (AFP-NHCC) patients, G-test alone had the greatest diagnostic capability (AUC = 0.855; P < 0.001), sensitivity (83.8%), and specificity (87.5%).

**Conclusion:**

G-test has the greatest diagnostic capability for HCC and AFP-NHCC, with high sensitivity and specificity, among healthy and LC patients. However, AAR had the highest diagnostic capability and sensitivity for HCC in CH. Overall, though, the combination of G-test, AFP and AAR provided the most optimal outcomes for predicting HCC onset, no matter the patient pre-conditions.

## Introduction

Hepatocellular carcinoma (HCC) is one of the leading causes of cancer-related death worldwide; its high morbidity and mortality rate has resulted in the cancer being considered a significant global health risk. Its occurrence is most commonly attributed to chronic viral hepatitis, alcohol intake and aflatoxin exposure. Owing to the lack of obvious symptoms at the early stages, most patients end up diagnosed with HCC after it has progressed to its advanced stages, contributing to its extremely low overall five-year survival rates of < 16% [1–4]. Currently, HCC is diagnosed based on a combination of serological, radiological, and pathological features, with liver biopsy being considered the gold standard; however, the procedure is limited by its invasiveness and risks for sampling errors [5]. As a result, alternative approaches for diagnosing HCC has been developed, such as imaging technologies, though this has its own shortcomings. For instance, it is difficult under conventional ultrasound to distinguish between benign and malignant small hepatocellular nodules [6, 7]. Thus, identifying a non-invasive, rapid, and easy-to-measure marker for HCC would be of great utility for clinical screening and development of more effective treatment approaches. The ideal biomarker is one that is easily detected in serum, plasma, bile, and other body fluids. Serum alpha fetoprotein (AFP) has long been considered as such a biomarker, but its usage, though widespread, has been controversial, owing to its low sensitivity to HCC, especially at its early stages, of 10–20%. This has led to its recommendation in HCC screening guidelines being highly controversial [1, 8]. An alternative proposed biomarker that has emerged is aspartate aminotransferase (AST) to alanine aminotransferase (ALT) ratio (AAR), which had already been used as an indicator of liver fibrosis. AAR has also been found to be able to serve as a biomarker for the identification and early prediction of HCC recurrence [9–11], leading to it being proposed as an effective marker for AFP-negative HCC (AFP-NHCC) [12]. More recently, changes in the N-glycome has been identified as a potential effective serum marker for liver disease. In particular, abnormal changes in the glycosylation modifications on glycoproteins have been observed throughout the progression of chronic liver disease into HCC [13–15]. This finding has led to the emergence of serum oligosaccharide chain (G-test) detection technology. G-test was first defined by Liu et al. [13] as the GlycoHCCTest, which was the log ratio of peak 9 to peak 7 obtained from DNA sequencer–assisted fluorophore-assisted carbohydrate electrophoresis of patient serum, followed by normal-phase high-performance liquid chromatography and digestion with exoglycosidases. The resulting peak 9 represented a branch α-(1,3)-fucosylated triantennary glycan, which was more abundant among HCC patients, compared to those without HCC. By contrast, peak 7, representing bisecting core α-(1,6)-fucosylated biantennary glycans, decreased with increasing stages of HCC. Therefore, higher G-test measurements correlated with HCC progression [13]. This finding was further verified in a previous study [16], in which higher G-test levels were associated with HCC onset among patients with chronic hepatitis B and related cirrhosis, and that G-test was better than AFP for predicting HCC. However, the effectiveness for G-test to diagnose HCC among broader patient demographics, as well as its predictive capabilities compared to other diagnostic markers, is still scarce.

In this study, we aim to fill in this knowledge gap by comparing the diagnostic value of G-test versus other markers. We examined the predictive capabilities of G-test, AFP, and AAR, both as single markers, and combined together, for detecting HCC in its early stages. We found that G-test had the greatest diagnostic ability for detecting HCC among healthy and liver cirrhosis (LC) patients, as well as AFP-NHCC among healthy, LC, and chronic hepatitis (CH) individuals. By contrast, AAR had the greatest diagnostic ability among CH. Regardless, the combination of all 3 tests yielded the most optimal outcomes with respect to diagnostic capability, sensitivity, and specificity for HCC.

## Methods

### Study design and population

Between Januarys 2020–2022, 152 subjects from the First Affiliated Hospital of Nanchang University were recruited and examined. HCC diagnoses among these subjects were based on histopathological analyses, according to the Guidelines of the American Association for the Study of Liver Diseases (AASLD) [17]. LC was diagnosed based on physical examinations, laboratory tests, as well as either B-mode ultrasound imaging or computed tomography (CT) of the liver. CH was diagnosed as stemming from a hepatitis B virus infection for more than 6 months, as well as presenting with persistent or intermittent elevated ALT and AST levels, along with chronic necrotic hepatitis tissue being present under liver biopsy [18]. Patients were excluded based on the following baseline criteria: (1) Absence of G-test, AFP, and/or AAR measurements, (2) Presence of other types of primary tumors, (3) Presence of other infectious diseases, such as HIV or non-HCC/LC/CH liver diseases (ex. drug-induced hepatitis, fatty liver, alcoholic liver disease, etc.), or (4) Presence of blood- or immune-related diseases. Application of the exclusion criteria yielded the aforementioned 152 subjects, of which 77 had HCC, 37 LC, 18 CH, and 20 served as healthy controls. The study protocol was approved by the Ethics Committee of the First Affiliated Hospital of Nanchang University. All patients provided written informed consent to participate in the study, and the study was conducted in full accordance with the Declaration of Helsinki.

### Data collection

All patient data was obtained from electronic medical records. Venous blood was collected after fasting and analyzed for the biochemical indices of AST, ALT, white blood cell (WBC) and platelet counts (PLT), as well as random blood glucose (RBG), total cholesterol (TC), triglyceride (TG), carcinoma embryonic antigen (CEA), total bilirubin (TBIL), albumin (ALB), prothrombin time (PT), AFP and G-test measurements. Most indices were measured using the Hitachi automatic biochemical analyzer (LABOSPECT008AS), though ALT and AST were determined by, respectively alanine and aspartate substrate method, with normal ranges defined as ~ 7–40 U/L for each. AAR was then calculated as AST/ALT. G-test levels were separated by fluorescence-labeled capillary micro-electrophoresis, with the positive standard for G-test being set at values > 5. AFP level was detected by electrochemiluminescence using the Roche automatic immune analyzer, with normal reference levels being ~ 0–7 ng/ml. AFP-NHCC was defined as AFP being < 20 µg/L, while AFP-positive HCC was defined as AFP ≥ 20 µg/L.

### Statistical analysis

SPSS 22.0 (SPSS Inc. Chicago, IL, USA), MedCalc v. 18.3.1 (MedCalc Software, Mariakerke, Belgium) and GraphPad Prism (version 8.0.2) software were used for all data analyses. The data for patient parameters were represented as mean ± standard deviation (SD) if it was normally-distributed, while non-normally distributed data was represented by medians (quartile). Classification data was represented as frequency and proportions. Differences between HCC, LC, CH, and healthy control groups for the different parameters were evaluated using either the non-parametric test for continuous variables, or χ2 test for categorical variables. Receiver operation characteristic (ROC) curves were then used to determine the areas under the curve (AUC), as well as the optimal cut-off values, sensitivity, specificity, plus positive and negative likelihood ratio (± LR) and predictive values (± PV), to determine the diagnostic values for AFP, G-test and AAR as predictive markers, either singly or in combination, for HCC occurrence. P < 0.05 was considered statistically significant for all analyses.

**Table 1:** Comparison of patient characteristics between non- and hepatocellular carcinoma (HCC) patient groups

Characteristics	Non-HCC			HCC	*p* value
	**Healthy**	**Chronic hepatitis**	**Liver Cirrhosis**		
Gender, male/female (n)	5/15	4/14	8/29	67/10	< 0.001
Age (years)	66.5 (48.3–75.8)	43.0 (31.5–50.5)	51.0 (42.0-65.5)	55.0 (47.5–67.0)	< 0.001
ALT (U/L)	17.3 (10.0–26.7)	132.5 (82.8-345.5)	37.4 (26.6–65.4)	31.0 (20.0-53.3)	< 0.001
AST (U/L)	25.4 (20.8–31.1)	70.3 (31.8–177.0)	45.9 (34.6–87.3)	42.5 (27.5–89.7)	< 0.001
TBIL (µmol/L)	15.3 (12.4–18.4)	78.5 (50.0-125.7)	25.4 (17.5–56.3)	20.4 (13.1–48.2)	< 0.001
ALB (g/L)	44.5 (43.0-45.8)	34.4 (29.6–35.0)	36.3 (30.3–61.9)	34.1 (28.1–37.4)	< 0.001
RBG (mmol/L)	5.23 (4.97–5.44)	4.64 (4.11–5.43)	4.81 (4.28–6.05)	4.80 (4.26–5.96)	< 0.001
TC (mmol/L)	5.26 (3.92–6.08)	3.65 (2.96–4.30)	3.42 (2.59–3.74)	3.89 (3.13–4.54)	< 0.001
TG (mmol/L)	0.98 (0.73–1.36)	0.87 (0.55–1.32)	0.61 (0.44–0.98)	0.88 (0.59–1.26)	0.002
PT (seconds)	NA	14.30 (12.30-16.45)	15.95 (14.30-17.48)	13.05 (11.90-15.15)	< 0.001
WBC count (×109/L)	5.39 (4.82–6.63)	5.45 (3.22–7.29)	3.73 (2.54–4.73)	4.45 (2.97–5.94)	0.007
Platelet count (×109/L)	234.0 (196.5-289.8)	172.0 (114.5-232.3)	67.0 (51.0-99.5)	91.0 (63.0-150.0)	< 0.001
AFP (ng/mL)	2.39 (1.77–3.95)	407.95 (84.90-817.35)	5.99 (2.05–44.82)	22.72 (2.92–608.70)	< 0.001
CEA (ng/mL)	1.88 (0.98–2.77)	3.08 (1.82–5.87)	2.67 (1.64–4.60)	2.93 (2.00-4.79)	0.011
AAR	1.44 (1.03–2.15)	0.58 (0.35–0.94)	1.35 (1.07–1.64)	1.40 (1.03–1.93)	< 0.001
G-Test	3.04 (1.54-4.00)	3.64 (2.77–5.12)	3.85 (2.59–6.03)	6.53 (5.87–7.38)	< 0.001

AAR: aspartate aminotransferase to alanine aminotransferase ratio; AFP: alpha-fetoprotein; ALB: albumin; ALT: alanine aminotransferase; AST: aspartate aminotransferase; CEA: carcinoma embryonic antigen; G-test: serum oligosaccharide chain; HCC: hepatocellular carcinoma; PT: prothrombin time; RBG: random blood glucose; TBIL: total bilirubin; TC: total cholesterol; TG: triglyceride; WBC: white blood cell count

## Results

### Clinical characteristics for the study subjects

**Fig. 1:**
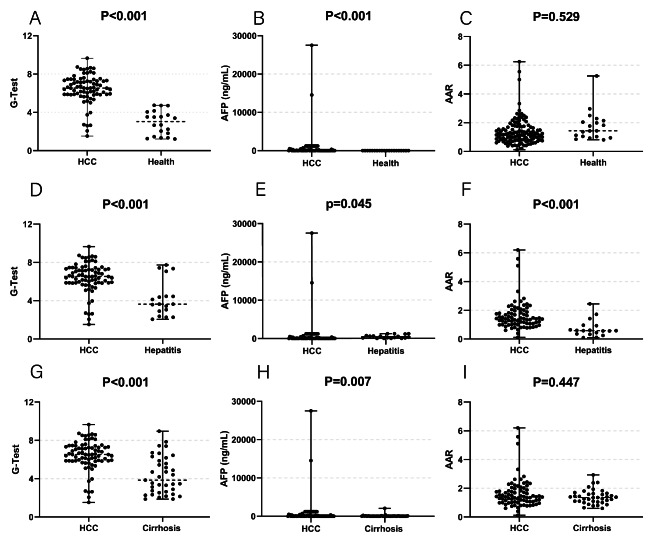
Comparisons between serum oligosaccharide chain (G-test), alpha-fetoprotein (AFP), and aspartate aminotransferase to alanine aminotransferase ratio (AAR) readings between **A-C** healthy, **D-F** chronic hepatitis (CH), and **G-I** liver cirrhosis (LC) groups versus hepatocellular carcinoma (HCC).

A total of 159 subjects were initially selected, of which 152 were analyzed in the final study. The remaining 7 were excluded for the following reasons: 2 for lack of clear diagnosis, 1 for having a liver abscess, 1 for drug-induced hepatitis, and 3 for lack of AFP or G-test results. As shown in Table 1, a greater proportion of male patients was present in the HCC group (87.0%), compared to CH (22.2%), LC (21.6%) or healthy control groups (25%). The median age for HCC was 55.0 years (47.5–67.0), higher than LC and CH, respectively at 51.0 (42.0-65.5) and 43.0 (31.5–50.5), but lower than the healthy control, at 66.5 (48.3–75.8). Positive AFP readings and G-test readings were observed in, respectively, 51.9% (40/77 patients), and 90.9% (70/77) of the HCC group, while negative AFP and G-test results were found among 64% (48/75) and 77.3% (58/75) of the non-HCC groups (Table 2). Figure 1 summarizes the comparison between G-test, AFP and AAR levels for HCC, versus healthy (Fig. 1 A-C), CH (Fig. 1D-F), and LC (Fig. 1G-I) patient groups. Statistically significant differences were found between HCC versus all 3 non-HCC groups for G-test and AFP. For AAR, significant differences were only present for HCC versus CH patients. Additionally, the HCC group had the highest G-test value (Table 1).

**Table 2:** Numbers of non- and HCC patients with AFP or G-test-negative/positive HCC

Variables		Non-HCC				HCC	Total
		**Healthy**	**Hepatitis**	**Cirrhosis**	**Total**		
AFP	+ (Positive)	0	14	13	27	40	67
	− (Negative)	20	4	24	48	37	85
	Total	20	18	37	75	77	152
G-Test	+ (Positive)	0	4	13	17	70	87
	− (Negative)	20	14	24	58	7	65
	Total	20	18	37	75	77	152

AFP: alpha-fetoprotein; G-test: serum oligosaccharide chain; HCC: hepatocellular carcinoma

### Comparing differences in sensitivity for HCC between different AFP thresholds

Table 3 shows the AFP levels for 77 patients in the HCC group, and different AFP threshold levels were established to determine its diagnostic capability for HCC, with the normal reference value set at 0–7 ng/mL. It was found that at AFP ≥ 7 ng/mL, 45 cases, or 58.4%, were diagnosed as HCC, but AFP sensitivity decreased to 41.6% at AFP ≥ 100 ng/mL. This sensitivity rate, however, was much greater than for the currently-recommended AFP diagnostic guidelines for liver cancer of ≥ 400 ng/mL. There, AFP sensitivity was only 27.3%, with missed diagnosis rate being as high as 72.7% (21/77).

**Table 3:** Percentages of correct and missed diagnoses for different biomarker thresholds in HCC group

Biomarker cutoffs	Positive/total cases	Rate of correct diagnosis (%)	Missed diagnosis rate (%)
G-Test	70/77	90.9	9.1
AFP ≥ 7	45/77	58.4	41.6
AFP ≥ 20	40/77	51.9	48.1
AFP ≥ 100	32/77	41.6	58.4
AFP ≥ 200	28/77	36.4	63.6
AFP ≥ 400	21/77	27.3	72.7

AFP: alpha-fetoprotein; G-test: serum oligosaccharide chain

### Evaluating the diagnostic values of the 3 biomarkers for HCC among different patient groups

**Fig. 2:**
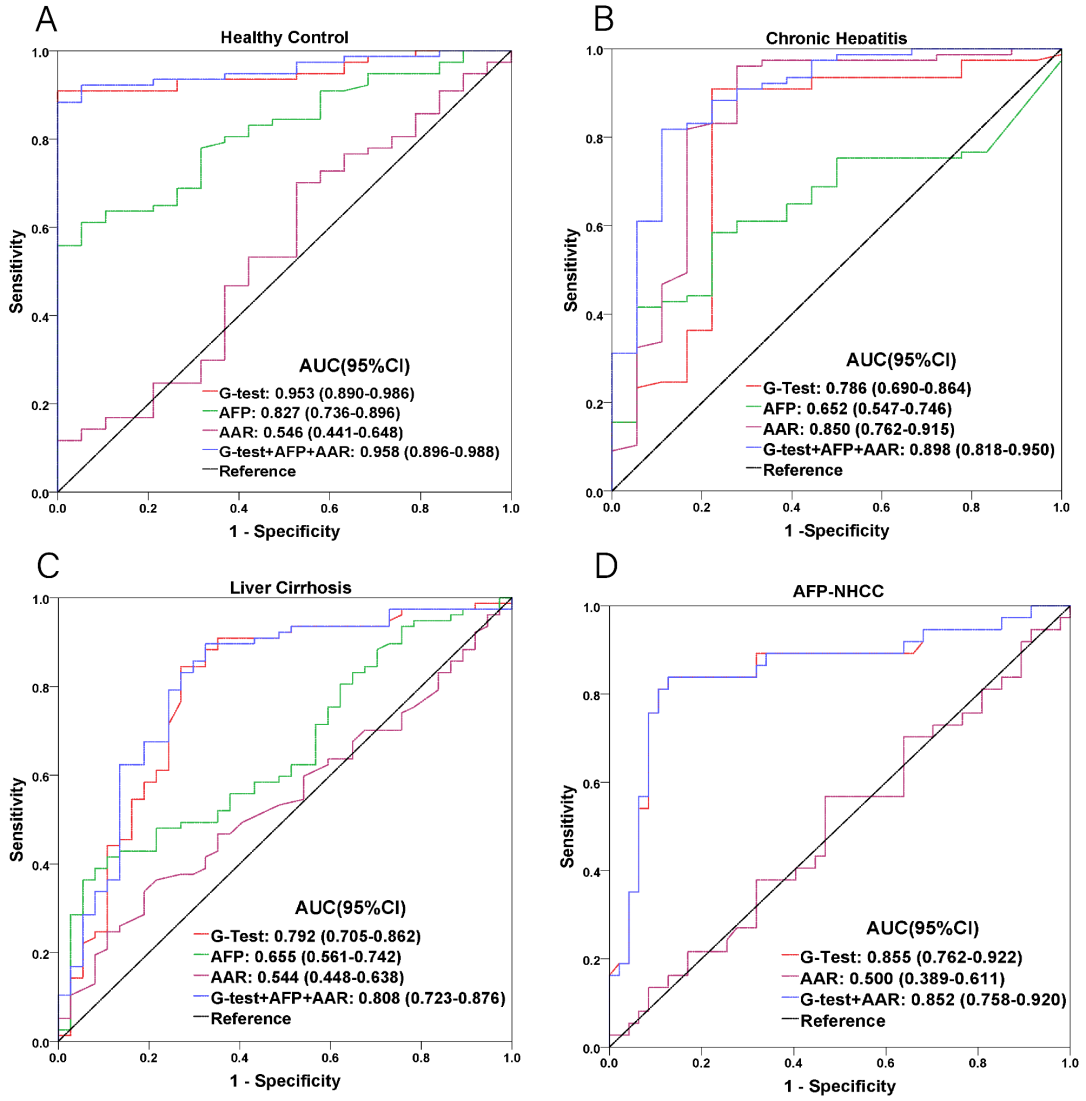
Receiver operating characteristic (ROC) curves for G-test, AFP, and AAR, both singly and in combination, among **A** healthy, **B** CH, and **C** LC with respect to HCC. **D** ROC curve for G-test and AAR, both singly and in combination, for AFP-negative HCC, compared to HCC.

To further evaluate the diagnostic value of G-test, AFP, and AAR, either alone or in combination, for HCC versus healthy controls, ROC curve analysis was used, as shown in Fig. 2 A. Out of the 3 diagnostic tests, G-test had the greatest diagnostic ability, with AUC of 0.953 (0.890–0.986), significantly higher than for AFP with 0.827 (0.736–0.896), and AAR with 0.546 (0.441–0.648). Additionally, G-test had the highest sensitivity and specificity, at 90.9% (82.2–96.3) and 100.0% (83.2–100.0), respectively (Table 4). The AUC for G-test, AAR and AFP combined was similar to that of G-test, at 0.958 (0.896–0.988), suggesting that G-test alone was as predictive for detecting HCC onset, compared to all 3 tests in combination, among healthy controls.

**Table 4:** Areas under the curve (AUC), sensitivity, and specificity measurements for G-test, AFP, and AAR (single and combination)

	AUC	Cut-offs	Sensitivity (%)	Specificity (%)	Youden index	+LR	-LR	+PV	-PV	*p* value
**Healthy vs. HCC**										
G-Test	0.953 (0.890–0.986)	4.73	90.9 (82.2–96.3)	100.0 (83.2–100.0)	0.909		0.091 (0.04–0.2)	100.0	74.1 (58.5–85.3)	< 0.001
AFP	0.827 (0.736–0.896)	5.28	61.0 (49.2–72.0)	95.0 (75.1–99.9)	0.560	12.2 (1.8–83.2)	0.4 (0.3–0.6)	97.9 (87.3–99.7)	38.8 (32.0–46.0)	< 0.001
AAR	0.546 (0.441–0.648)	1.75	70.1 (58.6–80.0)	47.4 (24.4–71.1)	0.175	1.3 (0.8–2.1)	0.63 (0.4–1.1)	84.4 (77.5–89.4)	28.1 (17.9–41.2)	0.552
G-Test + AFP + AAR	0.958 (0.896–0.988)	0.839	88.3 (79.0-94.5)	100.0 (82.4–100.0)	0.883		0.12 (0.06–0.2)	100.0	67.9 (53.3–79.6)	< 0.001
**Hepatitis vs. HCC**										
G-Test	0.786 (0.690–0.864)	4.47	90.9 (82.2–96.3)	77.8 (52.4–93.6)	0.687	4.1 (1.7–9.7)	0.1 (0.06–0.2)	94.6 (88.0-97.7)	66.7 (48.6–80.9)	< 0.001
AFP	0.652 (0.547–0.746)	96.14	58.4 (46.6–69.6)	77.8 (52.4–93.6)	0.362	2.6 (1.1–6.4)	0.5 (0.4–0.8)	91.8 (82.3–96.5)	30.4 (23.3–38.6)	0.013
AAR	0.850 (0.762–0.915)	1.53	96.1 (89.0-99.2)	72.2 (46.5–90.3)	0.683	3.5 (1.6–7.3)	0.1 (0.02–0.2)	93.7 (87.5–96.9)	81.2 (57.9–93.2)	< 0.001
G-Test + AFP + AAR	0.898 (0.818–0.950)	0.83	81.8 (71.4–89.7)	88.9 (65.3–98.6)	0.707	7.4 (2.0-27.3)	0.2 (0.1–0.3)	96.9 (89.5–99.2)	53.3 (40.9–65.4)	< 0.001
**Cirrhosis vs. HCC**										
G-Test	0.792 (0.705–0.862)	5.58	84.4 (74.4–91.7)	73.0 (55.9–86.2)	0.574	3.1 (1.8–5.3)	0.2 (0.1–0.4)	86.7 (79.1–91.8)	69.2 (56.3–79.7)	< 0.001
AFP	0.655 (0.561–0.742)	201.8	36.4 (25.7–48.1)	94.6 (81.8–99.3)	0.31	6.7 (1.7–26.7)	0.8 (0.6–0.8)	93.3 (77.9–98.2)	41.7 (37.2–46.2)	0.003
AAR	0.544 (0.448–0.638)	0.58	33.8 (23.4–45.4)	81.1 (64.8–92.0)	0.149	1.8 (0.9–3.7)	0.8 (0.7-1.0)	78.8 (64.0-88.6)	37.0 (32.0-42.4)	0.427
G-Test + AFP + AAR	0.808 (0.723–0.876)	0.62	89.6 (80.6–95.4)	67.6 (50.2–82.0)	0.572	2.8 (1.7–4.4)	0.2 (0.08–0.3)	85.2 (78.2–90.2)	75.8 (61.0-86.2)	< 0.001

±LR: positive/negative likelihood ratio; ±PV: positive/negative predictive value; AAR: aspartate aminotransferase to alanine aminotransferase ratio; AFP: alpha-fetoprotein; AUC: area under the curve; G-test: serum oligosaccharide chain; HCC: hepatocellular carcinoma

By contrast, the ROC curve analysis for G-test, AFP, and AAR among HCC versus CH patients found that AAR had the greatest diagnostic ability, with AUC of 0.850 (0.762–0.915), compared to 0.786 (0.690–0.864) and 0.652 (0.547–0.746) for, respectively, G-test and AFP (Fig. 2B). Furthermore, AAR had the highest sensitivity among the 3 tests, at 96.1% (89.0-99.2), but the lowest specificity, at 72.2% (46.5–90.3), compared to 77.8% (52.4–93.6) for G-test and AFP (Table 4). However, the diagnostic ability of all 3 tests combined was higher than for any of the tests alone, with AUC of 0.898 (0.818–0.950), as well as sensitivity at 81.8% (71.4–89.7) and specificity at 88.9% (65.3–98.6). Therefore, the combination of all 3 tests was determined to be the most optimal for detecting the presence of HCC among CH.

As for HCC versus LC patients, G-test was the most predictive for HCC occurrence, compared to AFP and AAR, with AUC of 0.792 (0.705–0.862) (Fig. 2 C). Additionally, G-test had the highest sensitivity, at 84.4% (78.4–91.7); however, AFP had the highest specificity, at 94.6% (81.8–99.3). All 3 tests combined, though, yielded the greatest diagnostic ability and highest sensitivity values, at respectively, AUC of 0.808 (0.723–0.876) and 89.6% (80.6–95.4) (Table 4). Therefore, the combination of all 3 tests was generally the most optimal for detecting HCC in LC, albeit it was slightly less specific in its detection, compared to AFP alone.

### Predictive capability, sensitivity, and specificity of the 3 biomarkers for AFP-NHCC

Among 152 subjects enrolled in the study, 85 were determined as being AFP-negative (Table 5), accounting for 70.6% of males. Out of those 85 patients, 37 patients (43.5%) were AFP-NHCC. ROC curve analysis was used to determine the diagnostic value for G-test and AAR, both separately and combined, for AFP-NHCC patients. The results showed that G-test had the highest diagnostic capability, with AUC of 0.855 (0.762–0.922), compared to 0.500 (0.389–0.611) for AAR (Fig. 2D). G-test also had the highest sensitivity and specificity, at 83.8% (68.0-93.8) and 87.5% (74.8–95.3), respectively. All these values for diagnostic capability, sensitivity, and specificity were similar for the combination of G-test and AAR versus G-test alone, indicating that G-test is sufficient for detecting AFP-NHCC.

**Table 5:** Areas under the curve (AUC), sensitivity, and specificity measurements for G-test and AAR (single and combination) between AFP-NHCC versus HCC

Variables	AUC	Cut-offs	Sensitivity (%)	Specificity (%)	Youden index	+LR	-LR	+PV	-PV	*p value*
G-Test	0.855 (0.762–0.922)	4.73	83.8 (68.0-93.8)	87.5 (74.8–95.3)	0.713	6.7 (3.1–14.4)	0.19 (0.09–0.4)	93.8 (70.7–91.7)	87.5 (77.0-93.6)	< 0.001
AAR	0.500 (0.389–0.611)	1.34	54.0 (36.9–70.5)	53.2 (38.1–67.9)	0.072	1.2 (0.8–1.8)	0.86 (0.6–1.3)	47.6 (37.3–58.2)	59.5 (48.6–69.6)	0.996
G-Test + AAR	0.852 (0.758–0.920)	0.44	83.8 (68.0-93.8)	87.2 (74.3–95.2)	0.710	6.6 (3.1–14.0)	0.19 (0.09–0.4)	83.8 (70.7–91.7)	87.2 (76.5–93.5)	< 0.001

±LR: positive/negative likelihood ratio; ±PV: positive/negative predictive value; AAR: aspartate aminotransferase to alanine aminotransferase ratio; AFP: alpha-fetoprotein; AFP-NHCC: alpha-fetoprotein negative hepatocellular carcinoma; AUC: area under the curve; G-test: serum oligosaccharide chain; HCC: hepatocellular carcinoma

## Discussion

HCC is one of the most common malignancies globally, with incidence increasing yearly by 3–9%, posing a huge global health risk. HCC patients typically have a low 5-year overall survival rate and poor clinical prognoses, due to the delay in diagnosing the disease. As a result, effective biomarkers have been long sought to increase HCC detection rates, for guiding individualized patient treatments [19, 20]. Some examples of biomarkers examined include G-test, AFP, and AAR. In this study, we also investigated these 3 biomarkers, both alone and in combination, to determine their predictive capabilities for diagnosing HCC. We found that G-test, AFP, and AAR levels were closely related to the occurrence of HCC, and that out of the 3 parameters, G-test had the greatest diagnostic value for predicting HCC, compared to AFP and AAR, as determined by AUC values. In particular, G-test had the highest specificity and sensitivity, compared to the other 2 markers, for LC and healthy individuals. However, for CH patients, AAR had the greatest sensitivity. Overall, though, the combination of all 3 biomarkers provided the most optimal sensitivity, specificity, and predictive capabilities for diagnosing HCC.

AFP was first proposed as a tumor marker for HCC in the 1960s and has since been widely used for clinical HCC detection. However, its utility for screening and diagnosis of HCC has been criticized, due to its low sensitivity and specificity [21–23]. It has been noted in multiple studies, though, that AFP sensitivity and specificity for HCC varied with the AFP threshold value; some studies showed that an AFP threshold of 20 ng/mL yielded sensitivity and specificity values for HCC detection of, respectively, 41-65% and 80-94% [24]. Increasing the AFP threshold from 20 to 50 ng/mL, though, yielded a significantly increased specificity value of 96%, along with a positive predictive value of 75%. On the other hand, sensitivity was only 47% [25]. Therefore, a lower AFP threshold value would yield increased sensitivity, but lower specificity, possibly leading to a greater risk for HCC false positives. These findings were in line with what was observed from 77 HCC patients in our study, in which at AFP thresholds of 7, 100, and 400 ng/mL, sensitivity for HCC was, respectively, 58.4%, 41.6% and 27.3%, while the rate of missed diagnosis increased from 41.6 to 72.7%. All these observations thus indicate the necessity of combining AFP with more effective biomarkers to obtain a better detection strategy for HCC. AAR has been used as an indicator to evaluate liver fibrosis in chronic liver disease [9, 26], and more recent studies have suggested that it can also be used to distinguish cirrhosis from HCC [10], with a sensitivity of 75.9% and specificity 55.7%. Additionally, AAR has been independently associated with early recurrence of HCC [11]. In this study, we evaluated the diagnostic ability of AAR, in terms of AUC, for HCC, and found that it had the highest value among CH patients, compared to G-test and AFP. This higher measurement is in line with AAR also having the greatest sensitivity among those patients. However, AAR, compared to G-test and AFP, had the lowest specificity for detecting HCC in CH.

Abnormal structural changes in liver glycosyltransferase have been noted as a key feature in the development of HCC, which could be reflected in the resulting serum N-glycan branching [27]. A study from Liu et al. [13] evaluated changes of the serum N-glycan spectrum among HCC patients using DNA sequencer-aided fluorophore-assisted carbohydrate electrophoresis, and the ratio of log (peak value 9/peak value 7 in the N-glycan spectrum) was named the GlycoHCCTest, or G-test for short. G-test has been found to be an effective and non-invasive means for detecting HCC among cirrhosis patients [28]. Our G-test results demonstrated that the test values was much higher among HCC, compared to CH and LC patients, which was consistent with the HCC developmental process progressing from hepatitis to cirrhosis to liver cancer experienced by most HCC patients. G-test was found by Wan et al. [16] to be superior to AFP in screening for liver cancer among patients with chronic hepatitis B and cirrhosis. Additionally, the combination of both parameters further improved the diagnosis rate for hepatitis B virus-related liver cancer. These findings were in line with our study, which evaluated a larger sample size and added AAR as a biomarker of interest, along with G-test, and AFP. We found that G-test was significantly better than AFP in distinguishing between those who developed HCC from those who did not, including healthy, CH, and LC individuals, while AAR was the most optimal only for differentiating between HCC and CH individuals. Nevertheless, the combination of G-test, AFP and AAR demonstrated the highest diagnostic capability, suggesting that this was likely the optimal approach for detecting HCC onset.

Cirrhosis and inflammation during HCC development complicate the early diagnosis of HCC. Due to the high rate of false negatives from AFP for HCC, biomarkers for AFP-NHCC have recently become a significant topic of interest. A study from Li et al. found that the gamma-glutamyl transpeptidase (GGT) to alkaline phosphatase ratio, combined with GGT to AST ratio and AAR, were effective diagnostic markers for AFP-NHCC [12]. In our study, we further analyzed the diagnostic ability of G-test and AAR for AFP-NHCC, and found that G-test, as well as the combination of G-test and AAR, was able to effectively detect AFP-NHCC. By contrast, AAR was significantly less effective for diagnosing AFP-NHCC. Furthermore, although both approaches used AAR levels as part of diagnosing HCC, our method was able to diagnose HCC onset using G-test and AFP, on top of AAR. The Li et al. method focused on detecting AFP-NHCC, while our method focused more on diagnosing HCC in general, being able to predict the occurrence of HCC [12], whether AFP-positive or negative (AFP-NHCC), among healthy, cirrhotic, and hepatitis (non-cancerous) patients. In addition, the Li et al. method was most effective during the early stages of AFP-NHCC, when the tumor size is small [12]. Nevertheless, additional studies are needed to unravel the true association between AAR and AFP-NHCC.

Changes in IgG antibody-linked oligosaccharides, in terms of both types and levels, have also been used as diagnostic markers for the onset and progression of various types of cancer. For instance, Kanoah et al. found that NSCLC progression was associated with significant decreases in mono- (Fr1) and digalactosyl (Fr2) IgG oligosaccharide levels, coupled with increases in agalactosyl IgG oligosaccharide (Fr4) [29]. Similar changes, entailing decreased Fr1 and F2, coupled with increased Fr4, were previously observed among that research group for prostate cancer [30]. More recently, changes in glycosylation patterns were observed in epithelial ovarian cancer patients, compared to healthy ones, with respect to IgG1, 2 and 3. In particular, IgG1 had significantly lower sialylation, and higher fucosylation, among cancer patients, while those patients also had increased agalactyosylation, along with decreased digalactosylation and sialylation, for IgG3. These alterations, especially for agalactosylation, were also found to be positively correlated with the widely utilized diagnostic marker CA125 [31]. However, the methods used to identify the oligosaccharide chains, such as fluorophore-associated carbohydrate electrophoresis and matrix-assisted laser desorption/ionization coupled with time-of-flight mass spectrometry are approaches that may be difficult to apply for widespread clinical use. Therefore, less cumbersome methods may need to be developed before IgG oligosaccharide chains could be utilized as a clinical diagnostic marker.

Moreover, it is worth noting that other methods for diagnosing HCC through serum diagnostic markers, such as exosomal DNA containing the TP53 gene mutation [32], phenylalanyl-tryptophan [33], miRNAs, such as miR-10b [34], prothrombin induced by vitamin K deficiency or antagonist-II [35], lncRNA-D16366[36], des-gamma-carboxyprothrombin [37], and dickkopf-1 [38], etc., have been documented. However, the widespread adoption for a number of these biomarkers as a diagnostic tool has been limited, owing to low sensitivity, which is exacerbated by HCC often being found alongside chronic liver disease and inflammation [39]. Furthermore, determining the appropriate cut-off values for detecting HCC onset with high specificity and sensitivity, as well as developing cost-effective approaches for measuring serum miRNA, lncRNA, and exosomal DNA levels, is a continued work in progress [39].

Our results provide a new predictor for diagnosing HCC, particularly AFP-NHCC. However, there are still a number of limitations in our study, one of which is its retrospective nature, which may reduce the predictive value of the results. Additionally, the sample size was small, possibly resulting in sampling biases. Lastly, there is a lack of sufficient HCC staging and follow-up data, limiting our ability to evaluate the association between the screening value of indicators with different HCC stages and follow-up findings. Therefore, future prospective studies with large sample sizes, multiple centers and adequate follow-up data collection are needed to validate the results.

## Conclusion

The biomarkers of G-test, AFP and AAR were examined as diagnostic indicators for HCC onset, and their predictive value was confirmed. Out of those 3 biomarkers, G-test had the highest predictive ability among healthy, LC, and AFP-NHCC patients, while AAR was the most predictive among CH. However, the combination of G-test, AFP and AAR has the highest diagnostic capability, sensitivity, and specificity for HCC, suggesting a possible new approach for screening and early detection of HCC. This finding thus facilitates interventions against HCC in its early stages among patient populations.

## Data Availability

All data generated or analyzed during this study are included in this published article.

## Abbreviations

±LR: positive/negative likelihood ratio.
±PV: positive/negative predictive value.
AAR: aspartate aminotransferase to alanine aminotransferase ratio.
AASLD: Association for the Study of Liver Diseases.
AFP: alpha-fetoprotein.
AFP-NHCC: alpha-fetoprotein negative hepatocellular carcinoma.
ALB: albumin.
ALT: alanine aminotransferase.
AST: aspartate aminotransferase.
AUC: area under the curve.
CEA: carcinoma embryonic antigen.
CH: chronic hepatitis.
CI: confidence intervals.
CT: computed tomography.
G-test: serum oligosaccharide chain.
GGT: gamma-glutamyl transpeptidase.
HCC: hepatocellular carcinoma.
LC: liver cirrhosis.
PLT: platelet count.
PT: prothrombin time.
RBG: random blood glucose.
ROC: Receiver operator characteristic.
SD: standard deviation.
TBIL: total bilirubin.
TC: total cholesterol.
TG: triglyceride.
WBC: white blood cell count.

## References

[1] Xing M, Wang X, Kiken RA, He L, Zhang JY. Immunodiagnostic Biomarkers for Hepatocellular Carcinoma (HCC): The First Step in Detection and Treatment. Int J Mol Sci. 2021 Jun 7;22(11):6139. doi: 10.3390/ijms22116139. 10.3390/ijms22116139 PMC8201127 34200243

[2] European Association for the Study of the Liver. Electronic address: easloffice@easloffice.eu; European Association for the Study of the Liver. EASL Clinical Practice Guidelines: Management of hepatocellular carcinoma. J Hepatol. 2018 Jul;69(1):182–236. doi: 10.1016/j.jhep.2018.03.019. Epub 2018 Apr 5. Erratum in: J Hepatol. 2019 Apr;70(4):817.

[3] Altekruse SF, McGlynn KA, Reichman ME. Hepatocellular carcinoma incidence, mortality, and survival trends in the United States from 1975 to 2005. J Clin Oncol. 2009 Mar 20;27(9):1485-91. doi: 10.1200/JCO.2008.20.7753. Epub 2009 Feb 17. 10.1200/JCO.2008.20.7753 PMC2668555 19224838

[4] Siegel R, Naishadham D, Jemal A. Cancer statistics. 2013. CA Cancer J Clin. 2013 Jan;63(1):11–30. doi: 10.3322/caac.21166. Epub 2013 Jan 17. 10.3322/caac.21166 23335087

[5] Zhao M, Hu Y, Shi C, Wang K, Li J, Song J, Huo C, Xi Y, Bu S, Huang Q. NFI, a clinical scoring tool for predicting non-alcoholic fatty liver in the Chinese population. Public Health. 2022 Jan;202:12–17. doi: 10.1016/j.puhe.2021.10.012. Epub 2021 Dec 4. 10.1016/j.puhe.2021.10.012 34875531

[6] Monfardini L, Orsi F, Caserta R, Sallemi C, Della Vigna P, Bonomo G, Varano G, Solbiati L, Mauri G (2018). Ultrasound and cone beam CT fusion for liver ablation: technical note. Int J Hyperthermia.

[7] Tsuchiya N, Sawada Y, Endo I, Saito K, Uemura Y, Nakatsura T. Biomarkers for the early diagnosis of hepatocellular carcinoma. World J Gastroenterol. 2015 Oct 7;21(37):10573-83. doi: 10.3748/wjg.v21.i37.10573. 10.3748/wjg.v21.i37.10573 PMC4588079 26457017

[8] Gao J, Song P (2017). Combination of triple biomarkers AFP, AFP-L3, and PIVAKII for early detection of hepatocellular carcinoma in China: Expectation. Drug Discov Ther.

[9] Giannini E, Botta F, Fasoli A, Ceppa P, Risso D, Lantieri PB, Celle G, Testa R (1999). Progressive liver functional impairment is associated with an increase in AST/ALT ratio. Dig Dis Sci.

[10] Giannini E, Risso D, Botta F, Chiarbonello B, Fasoli A, Malfatti F, Romagnoli P, Testa E, Ceppa P, Testa R (2003). Validity and clinical utility of the aspartate aminotransferase-alanine aminotransferase ratio in assessing disease severity and prognosis in patients with hepatitis C virus-related chronic liver disease. Arch Intern Med.

[11] Wang ZX, Jiang CP, Cao Y, Zhang G, Chen WB, Ding YT. Preoperative serum liver enzyme markers for predicting early recurrence after curative resection of hepatocellular carcinoma. Hepatobiliary Pancreat Dis Int. 2015 Apr;14(2):178–85. doi: 10.1016/s1499-3872(15)60353-8. 10.1016/s1499-3872(15)60353-8 25865691

[12] Li J, Tao H, Zhang E, Huang Z (2021). Diagnostic value of gamma-glutamyl transpeptidase to alkaline phosphatase ratio combined with gamma-glutamyl transpeptidase to aspartate aminotransferase ratio and alanine aminotransferase to aspartate aminotransferase ratio in alpha-fetoprotein-negative hepatocellular carcinoma. Cancer Med.

[13] Liu XE, Desmyter L, Gao CF, Laroy W, Dewaele S, Vanhooren V, Wang L, Zhuang H, Callewaert N, Libert C, Contreras R, Chen C (2007). N-glycomic changes in hepatocellular carcinoma patients with liver cirrhosis induced by hepatitis B virus. Hepatology.

[14] Dennis JW, Granovsky M, Warren CE. Glycoprotein glycosylation and cancer progression. Biochim Biophys Acta. 1999 Dec 6;1473(1):21–34. doi: 10.1016/s0304-4165(99)00167-1. 10.1016/s0304-4165(99)00167-1 10580127

[15] Ward DG, Cheng Y, N’Kontchou G, Thar TT, Barget N, Wei W, Billingham LJ, Martin A, Beaugrand M, Johnson PJ. Changes in the serum proteome associated with the development of hepatocellular carcinoma in hepatitis C-related cirrhosis. Br J Cancer. 2006 Jan 30;94(2):287–92. doi: 10.1038/sj.bjc.6602923. 10.1038/sj.bjc.6602923 PMC2361123 16404431

[16] Wan L, Guo L, Hu Y, Huang H, Zhang M, Xu K, De G, Zheng F, Wu Z, Hu C, Wen Z. Comparing the diagnostic value of serum oligosaccharide chain (G-test) and alpha-fetoprotein for hepatitis B virus-related liver cancer. Clin Biochem. 2021 Mar;89:44–50. doi: 10.1016/j.clinbiochem.2020.12.005. Epub 2020 Dec 10. 10.1016/j.clinbiochem.2020.12.005 33309517

[17] Heimbach JK, Kulik LM, Finn RS, Sirlin CB, Abecassis MM, Roberts LR, Zhu AX, Murad MH, Marrero JA. AASLD guidelines for the treatment of hepatocellular carcinoma. Hepatology. 2018 Jan;67(1):358–380. doi: 10.1002/hep.29086. 10.1002/hep.29086 28130846

[18] Lok AS, McMahon BJ. Chronic hepatitis B. Hepatology. 2007 Feb;45(2):507 – 39. doi: 10.1002/hep.21513. Erratum in: Hepatology. 2007 Jun;45(6):1347. PMID: 17256718. 10.1002/hep.21513 17256718

[19] Velázquez RF, Rodríguez M, Navascués CA, Linares A, Pérez R, Sotorríos NG, Martínez I, Rodrigo L. Prospective analysis of risk factors for hepatocellular carcinoma in patients with liver cirrhosis. Hepatology. 2003 Mar;37(3):520–7. doi: 10.1053/jhep.2003.50093. 10.1053/jhep.2003.50093 12601348

[20] Liu W, Chen Q, Mao M, Han R, Liu Y, Wang X. Novel prognostic scores based on serum ferritin/globulin ratio in patients with hepatocellular carcinoma. Transl Cancer Res. 2020 Oct;9(10):5925–5939. doi: 10.21037/tcr-20-966. 10.21037/tcr-20-966 PMC8797460 35117205

[21] Omata M, Cheng AL, Kokudo N, Kudo M, Lee JM, Jia J, Tateishi R, Han KH, Chawla YK, Shiina S, Jafri W, Payawal DA, Ohki T, Ogasawara S, Chen PJ, Lesmana CRA, Lesmana LA, Gani RA, Obi S, Dokmeci AK, Sarin SK. Asia-Pacific clinical practice guidelines on the management of hepatocellular carcinoma: a 2017 update. Hepatol Int. 2017 Jul;11(4):317–370. doi: 10.1007/s12072-017-9799-9. Epub 2017 Jun 15. 10.1007/s12072-017-9799-9 PMC5491694 28620797

[22] Kim DY, Han KH (2012). Epidemiology and surveillance of hepatocellular carcinoma. Liver Cancer.

[23] Hanif H, Ali MJ, Susheela AT, Khan IW, Luna-Cuadros MA, Khan MM, Lau DT (2022). Update on the applications and limitations of alpha-fetoprotein for hepatocellular carcinoma. World J Gastroenterol.

[24] Gupta S, Bent S, Kohlwes J (2003). Test characteristics of alpha-fetoprotein for detecting hepatocellular carcinoma in patients with hepatitis C. A systematic review and critical analysis. Ann Intern Med.

[25] Gambarin-Gelwan M, Wolf DC, Shapiro R, Schwartz ME, Min AD (2000). Sensitivity of commonly available screening tests in detecting hepatocellular carcinoma in cirrhotic patients undergoing liver transplantation. Am J Gastroenterol.

[26] Williams AL, Hoofnagle JH (1988). Ratio of serum aspartate to alanine aminotransferase in chronic hepatitis. Relationship to cirrhosis. Gastroenterology.

[27] Zhao YY, Takahashi M, Gu JG, Miyoshi E, Matsumoto A, Kitazume S, Taniguchi N. Functional roles of N-glycans in cell signaling and cell adhesion in cancer. Cancer Sci. 2008 Jul;99(7):1304–10. doi: 10.1111/j.1349-7006.2008.00839.x. Epub 2008 May 19. 10.1111/j.1349-7006.2008.00839.x PMC11158068 18492092

[28] Liu Y, He J, Li C, Benitez R, Fu S, Marrero J, Lubman DM. Identification and confirmation of biomarkers using an integrated platform for quantitative analysis of glycoproteins and their glycosylations. J Proteome Res. 2010 Feb 5;9(2):798–805. doi: 10.1021/pr900715p. 10.1021/pr900715p PMC2838716 19961239

[29] Kanoh Y, Ohara T, Mashiko T, Abe T, Masuda N, Akahoshi T (2006). Relationship between N-linked oligosaccharide chains of human serum immunoglobulin G and serum tumor markers with non-small cell lung cancer progression. Anticancer Res.

[30] Kanoh Y, Mashiko T, Danbara M, Takayama Y, Ohtani S, Egawa S, Baba S, Akahoshi T (2004). Changes in serum IgG oligosaccharide chains with prostate cancer progression. Anticancer Res.

[31] Wieczorek M, Braicu EI, Oliveira-Ferrer L, Sehouli J, Blanchard V, Immunoglobulin G (2020). Subclass-Specific Glycosylation Changes in Primary Epithelial Ovarian Cancer. Front Immunol.

[32] Li Y, Wu J, Li E, Xiao Z, Lei J, Zhou F, Yin X, Hu D, Mao Y, Wu L, Wenjun L (2022). TP53 mutation detected in circulating exosomal DNA is associated with prognosis of patients with hepatocellular carcinoma. Cancer Biol Ther.

[33] Luo P, Yin P, Hua R, Tan Y, Li Z, Qiu G, Yin Z, Xie X, Wang X, Chen W, Zhou L, Wang X, Li Y, Chen H, Gao L, Lu X, Wu T, Wang H, Niu J, Xu G (2018). A Large-scale, multicenter serum metabolite biomarker identification study for the early detection of hepatocellular carcinoma. Hepatology.

[34] Aksoy F, Ak Aksoy S, Dundar HZ, Tunca B, Ercelik M, Tekin Ç, Kıyıcı M, Selimoglu K, Kaya E. Blood-Based Biomarkers in Afp Normal/Stable Hepatocellular Carcinoma: Diagnostic and Prognostic Relevance of Mir-10b for Patients on Liver Transplant List. Transplant Proc. 2022 Aug 17:S0041-1345(22)00451-1. 10.1016/j.transproceed.2022.05.024 35987859

[35] Feng H, Li B, Li Z, Wei Q, Ren L. PIVKA-II serves as a potential biomarker that complements AFP for the diagnosis of hepatocellular carcinoma. BMC Cancer. 2021 Apr 13;21(1):401. 10.1186/s12885-021-08138-3 PMC8045263 33849479

[36] Chao Y, Zhou D (2019). lncRNA-D16366 Is a Potential Biomarker for Diagnosis and Prognosis of Hepatocellular Carcinoma. Med Sci Monit.

[37] Liu Z, Wu M, Lin D, Li N (2020). Des-gamma-carboxyprothrombin is a favorable biomarker for the early diagnosis of alfa-fetoprotein-negative hepatitis B virus-related hepatocellular carcinoma. J Int Med Res.

[38] Kim SU, Park JH, Kim HS, Lee JM, Lee HG, Kim H, Choi SH, Baek S, Kim BK, Park JY, Kim DY, Ahn SH, Lee JD, Han KH (2015). Serum Dickkopf-1 as a Biomarker for the Diagnosis of Hepatocellular Carcinoma. Yonsei Med J.

[39] Piñero F, Dirchwolf M, Pessôa MG. Biomarkers in Hepatocellular Carcinoma: Diagnosis, Prognosis and Treatment Response Assessment. Cells. 2020 Jun 1;9(6):1370. 10.3390/cells9061370 PMC7349517 32492896

